# Complete Genome Sequence of Lytic Oenococcus oeni Bacteriophage OE33PA

**DOI:** 10.1128/MRA.00818-18

**Published:** 2018-08-16

**Authors:** Fety Jaomanjaka, Olivier Claisse, Cécile Philippe, Claire Le Marrec

**Affiliations:** a ISVV, EA4577 Oenologie, University of Bordeaux, Villenave d’Ornon, France; b INRA, ISVV, USC 1366 Oenologie, Villenave d’Ornon, France; c Bordeaux INP, ISVV, EA4577 Oenologie, Villenave d’Ornon, France; Georgia Institute of Technology

## Abstract

Oenococcus oeni is the most common species of lactic acid bacteria associated with malolactic fermentation in wine. Here, we report the genome sequence of the lytic phage OE33PA (vB_OeS_OE33PA). It has a morphotype similar to that of members of the Siphoviridae family, a linear 39,866-bp double-stranded genome with cohesive ends, and 57 predicted open reading frames.

## Abstract (precis)

Oenococcus oeni is the most common species of lactic acid bacteria associated with malolactic fermentation in wine. Here, we report the genome sequence of the lytic phage OE33PA (vB_OeS_OE33PA).

## ANNOUNCEMENT

After alcoholic fermentation, most wines undergo malolactic fermentation (MLF), driven by Oenococcus oeni, to improve their organoleptic properties and microbiological stability (1). Phages infecting O. oeni have been examined, as they could delay MLF (2–6) and open the way for less-desired indigenous populations to dominate in wines, impairing quality (1, 7). However, so far, no free phage infecting O. oeni has been sequenced, and our current knowledge about phage genomes is derived from the sequencing of prophage loci recognized in prokaryotic genome sequencing projects (8, 9).

Phage OE33PA (vB_OeS_OE33PA) was recently isolated from a red wine using O. oeni host strain IOEBS277 (10). Its apparent obligately lytic lifestyle was intriguing since most oenophages reported to date are temperate (11). Purified DNA was sequenced using Illumina MiSeq technology (Genome-Transcriptome facility of Bordeaux). An average coverage of 3,855× was achieved. Reads were assembled into a single contig using SPAdes 3.10.1 (12), and phage genome ends were determined through closure PCR and Sanger sequencing.

Phage OE33PA has a linear double-stranded DNA genome of 39,866 bp with 13-base-long cohesive ends (CGCACACATTGGA) and a G+C content of 37.29%. Annotation of the open reading frames (ORFs) was performed using Prokka v1.12 (13) with a custom database containing phage sequences from the Swiss-Prot database and refined with the Rapid Annotation using Subsystems Technology server (14). A total of 57 ORFs were predicted from the genome, and no tRNAs were found. All ORFs translate into proteins ranging in size from 44 to 1,613 amino acids. A total of 49 ORFs are predicted on the forward strand, while the remaining 8 ORFs have a reverse orientation.

Sequence similarity searches were performed with the translation of each predicted ORF against the NCBI protein database using BLASTp (15). Based on homology to known phage proteins, 28 out of 57 ORFs were assigned a predicted function. Although OE33PA cannot lysogenize its host (10), it is predicted to contain a typical lysogeny module, suggesting that a temperate ancestor spontaneously gave rise to the virulent OE33PA mutant. The lysogeny module includes six genes which are all divergently transcribed, of which two encode hypothetical proteins with a short C-terminal (SHOCT) domain (16). The module also encodes a repressor and an integrase which shares 99% identity with that of oenophage Φ10MC (17). However, the 15-bp *attP* site found directly downstream of the *int* gene in Φ10MC was not identified in OE33PA (17).

The genome of OE33PA contains functional genes related to replication (helicases, primase), structure proteins (several tail proteins, phage head-tail adaptor protein), packaging (terminase and portal protein), and lysis (lysin and holin). Two extra nonphage genes (morons) downstream of the lysis cassette specify a putative sulfite exporter and a glycosyltransferase.

### Data availability.

The phage genome sequence is available at GenBank under the accession no. MH220877.
